# Effects of (1*E*,4*E*)-2-Methyl-1,5-bis(4-nitrophenyl)penta-1,4-dien-3-one on* Trypanosoma cruzi* and Its Combinational Effect with Benznidazole, Ketoconazole, or Fluconazole

**DOI:** 10.1155/2017/7254193

**Published:** 2017-05-23

**Authors:** Francieli Peron, Danielle Lazarin-Bidóia, Zia Ud Din, Edson Rodrigues-Filho, Tânia Ueda-Nakamura, Sueli de Oliveira Silva, Celso Vataru Nakamura

**Affiliations:** ^1^Programa de Pós-Graduação em Ciências Biológicas, Universidade Estadual de Maringá, Av. Colombo 5790, 87020-900 Maringá, PR, Brazil; ^2^Programa de Pós-Graduação em Ciências Farmacêuticas, Universidade Estadual de Maringá, Av. Colombo 5790, 87020-900 Maringá, PR, Brazil; ^3^LaBioMMi, Departamento de Química, Universidade Federal de São Carlos, CP 676, 13.565-905 São Carlos, SP, Brazil

## Abstract

This study reports the activity induced by (1*E*,4*E*)-2-methyl-1,5-bis(4-nitrophenyl)penta-1,4-dien-3-one** (A3K2A3)** against* Trypanosoma cruzi.* This compound showed trypanocidal activity against the multiplicative epimastigote and amastigote forms of this protozoan, with IC_50_ values of 1.99 ± 0.17 and 1.20 ± 0.16 *μ*M, respectively, and EC_50_ value of 15.57 ± 0.34 *μ*M against trypomastigotes. The combination of** A3K2A3** with benznidazole or ketoconazole demonstrated strong synergism, increasing effectiveness against trypomastigotes or epimastigotes of* T. cruzi*. In addition, the drug combination of** A3K2A3 **with benznidazole or ketoconazole on LLCMK_2_ cells demonstrated an antagonist effect, which resulted in greater protection of the cells from drug damage. The combination of the compound with fluconazole was not effective. Transmission and scanning electron micrographs showed changes on parasites, mainly in the cytoplasmatic membrane, nucleus, mitochondrion, and Golgi complex, and a large increase in the number of autophagosome-like structures and lipid-storage bodies, accompanied by volume reduction and rounding of the parasite.** A3K2A3** might be a promising compound against* T. cruzi*.

## 1. Introduction

Chagas disease was discovered and described in 1909 by the Brazilian physician Carlos Chagas. It is considered a public health problem in 19 American countries and about 7 to 8 million people are infected worldwide, especially in Latin America. Also, several cases of the disease have been detected in United States, Canada, Europe, and some countries of the Western Pacific [1–3].

The causative agent of Chagas disease,* Trypanosoma cruzi*, presents heteroxenic life cycle, involving a mammalian host and an invertebrate host (triatomine) and three main morphologically distinct evolutionary forms can be highlighted, epimastigote, trypomastigote, and amastigote [4].

Chagas disease presents itself in 2 phases: the acute phase and the chronic phase. The initial, acute phase lasts for 4–8 weeks after infection and is usually asymptomatic or could present as a self-limiting febrile illness. Additionally, some individuals can present headache, enlarged lymph glands, pallor, muscle pain, difficulty in breathing, swelling, and abdominal or chest pain. However in several cases there is still morbidity and mortality [5]. In the second, chronic stage, the infection may remain silent for decades or even for life. However, some patients suffer from cardiac disorders and digestive, neurological, or mixed complications. This infection can lead to sudden death or heart failure caused by progressive destruction of the heart muscle and of the nervous system [1].

The treatment is restricted to only two nitroheterocyclic compounds, benznidazole and nifurtimox, developed in the 70s [6]. Both drugs are far from ideal, due to the limited effectiveness against different strains of the parasite, the need for long-term therapy, its weak activity in the chronic phase, and systemic toxicity with serious side effects, including polyneuritis, lymphadenopathy, dermatitis, anorexia, allergic dermopathy, and depression of bone marrow [7]. Because of these adverse effects, many patients end up abandoning the treatment [8]. These drawbacks justify the need to identify new drugs that are more effective and less toxic for the treatment of patients with Chagas disease.

Several studies have been conducted to find new active compounds against* T. cruzi*. The search based on natural and synthetic compounds is promising, either alone or in combination, against the parasite [9–12]. It is proven that therapeutic associations are an important way of increasing efficacy and reduce the toxicity of drugs with different mechanisms of action in the treatment of many diseases [13]. Previous works have shown the importance of combination therapy against several microorganisms such as bacteria [14, 15], fungi [16, 17], and protozoa such as* Leishmania* [18, 19] and* T. cruzi *[9, 20, 21].

Dibenzylideneacetone (DBA) is a class of compounds having an acyclic dienone attached to aryl groups in both *β*-positions. These structures are similar to chalcones and curcuminoids, which are important bioactive natural compounds found in several plant species. DBAs have shown antiplasmodial [22], antitumor [23], and anticancer activity inhibiting cell growth and inducing apoptosis [24]. Additionally, DBA potentiates TRAIL-induced apoptosis through downregulation of cell survival proteins and upregulation of death receptors via activation of reactive oxygen species (ROS) [25]. Recently, our research group reported trypanocidal activity of a DBA (1*E*,4*E*)-2-methyl-1,5-bis(4-nitrophenyl)penta-1,4-dien-3-one** (A3K2A3)** against epimastigote and trypomastigote forms of* T. cruzi* [26].

Considering the trypanocidal activity of this compound, the aim of this study was to further evaluate the antiprotozoan activity and the main morphological and ultrastructural alterations induced by** A3K2A3**, against the three main forms of* T. cruzi*, and investigate the safety of these compounds, testing their toxicity in human red blood cells. We also verified the in vitro activity of** A3K2A3** combined with benznidazole, ketoconazole, and fluconazole against* T. cruzi* and mammalian cells.

## 2. Materials and Methods

### 2.1. Synthesis of the Compound** A3K2A3**

**Figure 1:**
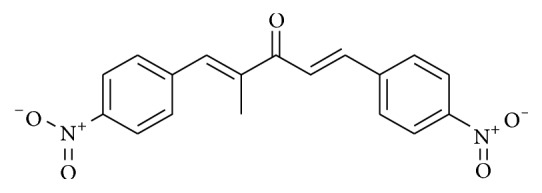
Structure of dibenzylideneacetone (1*E*,4*E*)-2-methyl-1,5-bis(4-nitrophenyl)penta-1,4-dien-3-one.

The compound (1*E*,4*E*)-2-methyl-1,5-bis(4-nitrophenyl)penta-1,4-dien-3-one** (A3K2A3)** (Figure 1) is dibenzylideneacetone synthesized following the methodology described by Din et al. [26]. Stock solution of the compound was prepared aseptically in DMSO and diluted in culture medium so that the DMSO concentration did not exceed 1% in the experiments.

### 2.2. Parasites and Mammalian Cells

Epimastigote forms of* Trypanosoma cruzi* (Y strain) were cultivated in Liver Infusion Tryptose (LIT) medium [27] supplemented with 10% heat-inactivated fetal bovine serum (FBS; Gibco Invitrogen, Grand Island, NY, USA), kept at 28°C, and maintained by weekly transfers. Trypomastigote forms were obtained from the supernatant of previously infected monolayers of LLCMK_2_ cells (*Macaca mulatta *epithelial kidney cells; American Type Culture Collection, Rockville, MD, USA) in DMEM supplemented with 2 mM L-glutamine, 10% FBS, and 50 mg/L gentamicin and buffered with sodium bicarbonate in a 5% CO_2_ air mixture at 37°C.

### 2.3. Antiproliferative Assay

Epimastigotes (1.0 × 10^6^ parasites/mL) in the exponential phase of growth (96 h) were incubated in LIT medium plus 10% FBS and added increasing concentrations of** A3K2A3** (1–100 *μ*M) in 24-well plates. Parasites were incubated at 28°C and collected aseptically every 24 h and counted in a Neubauer hemocytometer. The IC_50_ (concentration that inhibited 50% of parasite growth) were determined by regression analysis of the data.

### 2.4. Viability Assay in Trypomastigote Forms

Trypomastigotes (1.0 × 10^7^ parasites/mL) were incubated in 96-well plates containing 20% mouse blood or not and 20% FBS and added increasing concentrations of** A3K2A3** (1–100 *μ*M). Parasites were incubated for 24 h at 4°C or 37°C in 5% CO_2_ atmosphere. After incubation, the viability of the parasites was examined by mobility under a light microscope (Olympus CX31) using the Pizzi-Brener method [28]. The concentration that killed 50% of the parasites (EC_50_) was calculated.

### 2.5. Antiproliferative Assay in Intracellular Amastigotes

LLCMK_2_ cells (2.5 × 10^5^ cells/mL) were added to 24-well plates that contained round glass coverslips and incubated at 37°C in 5% CO_2_ atmosphere until confluent growth (24 h). After, the cells were infected with trypomastigote forms (10 : 1 ratio) during 24 h and washed in phosphate-buffered saline (PBS). DMEM with increasing concentrations of** A3K2A3** (1–100 *μ*M) was added and the cells were incubated under the same conditions for a period of 96 h. Then, the glass coverslips were subjected to fixation with methanol and stained with May-Grunwald Giemsa for 20 min and permanently prepared with Entellan (Merck, Germany). The percentage of infected cells and the number of parasites per cell were determined by counting 200 cells under a light microscope. The survival index (%) was obtained by multiplying the percentage of infected cells by the mean number of amastigotes per infected LLCMK_2_ cell. The survival index observed in the control without treatment was considered as 100% and the results for treated groups were comparatively evaluated.

### 2.6. Hemolytic Activity

The lytic activity of the** A3K2A3 **on human red blood cells evaluated using erythrocytes from a healthy human donor was obtained. For this, cells were defibrinated, washed in glycosylated saline, resuspended to 3% in glycosylated saline, and incubated with increasing concentrations of the** A3K2A3** (1–1000 *μ*M) at 37°C for 3 h. After, the supernatant was read in microplate (Biotek PowerWave XS) at 550 nm. Triton X-100 (1%) was used as a positive control.

The blood was collected by brachial vein puncture from healthy volunteer donors agreeing with Declaration of Helsinki, a set of ethical principles for medical research involving human subjects, last reviewed in 2013. All procedures were performed according to specific protocol approved by State University of Maringá (acceptance 293/2006 COPEP-UEM).

### 2.7. Drug Combination

To verify the effect of the combination of** A3K2A3** associated with benznidazole, fluconazole, or ketoconazole on epimastigotes, trypomastigotes, and LLCMK_2_ cells, we applied the Combination Index (CI) method proposed by Chou and Talalay [29] and revised by Zhao et al. [30]. The experimental design consists of combinations of at least four concentrations of each drug arranged in a checkerboard at a 1 : 2 concentration ratio. Briefly, epimastigotes (1.0 × 10^6^ parasites/mL) were resuspended in LIT medium in the presence of different concentrations of each drug in combination and counted after 96 h incubation at 28°C. Trypomastigotes (1.0 × 10^7^ parasites/mL) were collected and exposed to different concentrations of drugs in combination and after 24 h incubation, the parasites were analyzed for viability. LLCMK_2_ cells (2.5 × 10^5^ cells/mL) were plated and exposed to different concentrations of drugs in combination. After 96 h, cell viability was quantified using the MTT method [31]. The data were calculated and mathematically expressed as the Combination Index (CI = [IC_50_ drug A combined/IC_50_ drug A alone] + [IC_50_ drug B combined/IC_50_ drug B alone]), the numerators represent the combined concentration of each drug that inhibited 50% of the growth of the cells, the denominator is the concentration of each drug alone that exhibited the same effect. The resulting CI theorem of Chou-Talalay offers quantitative definition for additive (CI = 1), synergism (CI < 1), and antagonism (CI > 1) effects in drug combinations. The data were also graphically expressed as isobolograms.

### 2.8. Scanning Electron Microscopy (SEM)

To evaluate the effect of the** A3K2A3** on the morphology of the parasite by SEM, parasitic forms and LLCMK_2_ cells were treated at the concentrations that corresponded to the IC_50_ and IC_90_ (epimastigotes and amastigotes for 72 h) or EC_50_ and EC_90_ (trypomastigotes for 24 h). The parasites were collected, washed, and fixed in 2.5% glutaraldehyde in 0.1 M sodium cacodylate buffer at 4°C for 24 h. After, they were added onto glass coverslips containing poly-L-lysine, dehydrated in increasing gradient of ethanol, dried by the critical-point method using CO_2_, coated with gold, and observed in Shimadzu SS-550 SEM.

In the images obtained by SEM, protozoa were analyzed for changes in their cell surface, number and flagella morphology, leakage of cytoplasmic contents, and size and cell shape changes.

### 2.9. Transmission Electron Microscopy (TEM)

To evaluate the effect of the** A3K2A3** on the ultrastructure of the parasite by TEM, treated parasitic forms were collected and fixed under the same conditions as the SEM. The postfixation was performed in a solution of 1% OsO_4_, 0.8% potassium ferricyanide, and 10 mM CaCl_2_, dehydrated in acetone, and embedded in Polybed 812 resin. Ultrathin sections were obtained, contrasted with uranyl acetate and lead citrate, and observed in JEOL JM 1400 TEM.

### 2.10. Statistical Analysis

All of the quantitative experiments were performed in at least three independent experiments in duplicate. The statistical analyses were performed using GraphPad Prism® Software (GraphPad Software, California, USA). The data were analyzed using one-way analysis of variance (ANOVA), and the Tukey post hoc test was used to compare means when appropriate. Values of *p* ≤ 0.05 were considered statistically significant.

## 3. Results

### 3.1. **A3K2A3** Inhibits the Proliferation of Epimastigote Forms of* Trypanosoma cruzi*

**Figure 2:**
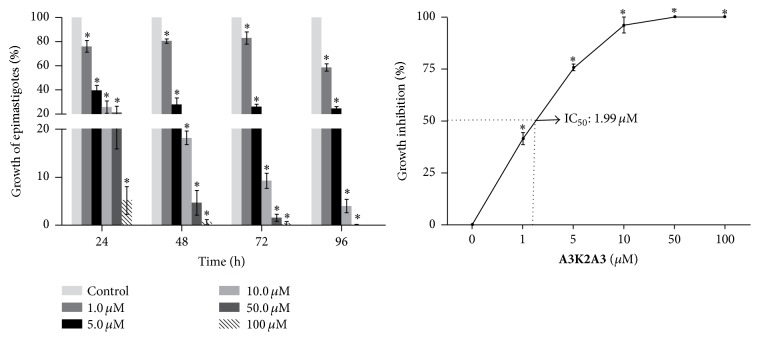
Antiproliferative activity of** A3K2A3** against the epimastigote forms of* T. cruzi*. The parasite was incubated in the absence or presence of different concentrations of** A3K2A3**, and cell growth was analyzed 24, 48, 72, and 96 h after treatment. The results are expressed as percentages ± SD of growth compared to untreated parasites of at least three independent experiments. The dotted line represents the IC_50_ value after 96 h of treatment. ^*∗*^*p* ≤ 0.05, significant difference relative to the control group (untreated parasites).

The compound** A3K2A3** showed activity against all three forms of* Trypanosoma cruzi*. In epimastigotes the compounds caused a dose-dependent inhibition of growth of protozoa, exhibiting an IC_50_ of 1.99 ± 0.17 *μ*M in the treatment for 96 h. By monitoring the number of parasites every 24 h, we noticed that a large proportion of the effect was reached after 24 h incubation (Figure 2). It is important to note that the activity of the compound was more effective than the standard drug benznidazole (IC_50_ of 6.4 *μ*M) [10].

### 3.2. **A3K2A3 **Decreases the Viability of Trypomastigote Forms of* Trypanosoma cruzi*

**Figure 3:**
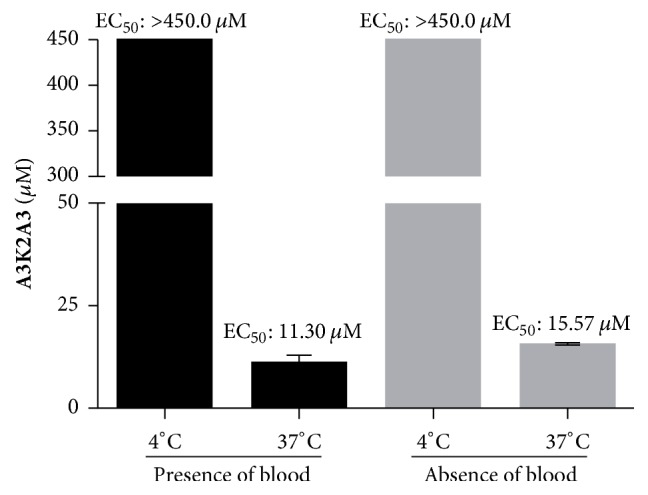
Activity of** A3K2A3** against trypomastigote forms of* T. cruzi*. Effective concentration (EC_50_) of** A3K2A3** that inhibited the viability of 50% of the trypomastigotes after 24 h incubation, assays conducted with presence or absence of blood at 4°C and 37°C. Results are expressed as mean ± SD of at least three independent experiments.

The effect of compound** A3K2A3** was evaluated against trypomastigotes in experiments with and without addition of blood and at two different temperatures. The results (Figure 3) showed a marked reduction in the activity of the compounds in the presence and absence of blood and at 4°C (EC_50_ > 450.0 *μ*M) when compared to the effects of the compound at 37°C.** A3K2A3** exhibited a slightly more pronounced effect on trypomastigotes in the presence of blood at 37°C (EC_50_  11.3 ± 1.17 *μ*M) when compared to the same assay, but in the absence of blood (EC_50_  15.57 ± 0.34 *μ*M).

### 3.3. **A3K2A3** Induces Trypanocidal Effect on Amastigote Forms of* Trypanosoma cruzi*

**Figure 4:**
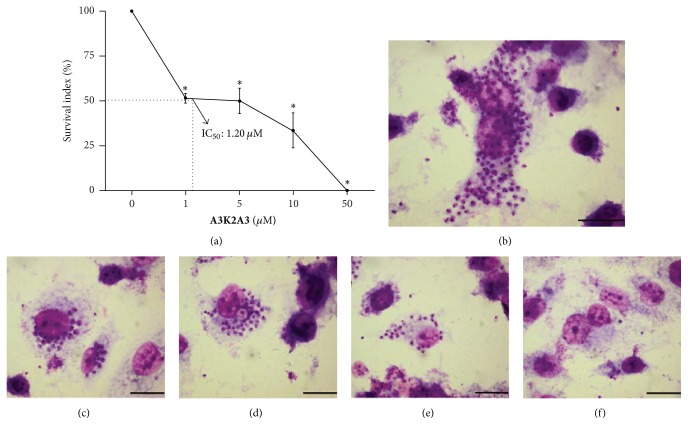
Antiproliferative activity of** A3K2A3** against intracellular amastigote forms of* T. cruzi* after 96 h of treatment. (a) LLCMK_2_ cells were infected with trypomastigotes and then treated with** A3K2A3**. The data are expressed as the mean values of at least three independent experiments. The values obtained for the survival index of the control group (untreated cells) were normalized to 100%. The dotted line represents the IC_50_ value after 96 h of treatment. ^*∗*^*p* ≤ 0.05, significant difference of each group from control. Light microscopy of* T. cruzi*-infected LLCMK_2_ cells treated for 96 h. (b) Controls cells. (c, d, e, f) Cells treated with 1.0, 5.0, 10.0, and 50.0 *μ*M of** A3K2A3**, respectively. Bars = 20.0 *µ*m.

**A3K2A3** also exerted inhibitory effect against intracellular amastigotes. After 96 h incubation in presence of the compound, the percentages of infected LLCMK_2_ cells and mean number of intracellular amastigotes per infected cells were reduced in comparison with the untreated control (Figure 4). Both parameters are expressed together as the survival rate.** A3K2A3** compound inhibited the growth of 50% of amastigotes at concentration 1.20 ± 0.16 *μ*M. Additionally,** A3K2A3** showed no toxicity in mammalian LLCMK_2_ cells with 50.0 *μ*M, a concentration that completely reduced the proliferation of intracellular amastigotes (Figure 4(f)).

### 3.4. **A3K2A3** Does Not Cause Toxicity in Red Blood Cells

**Figure 5:**
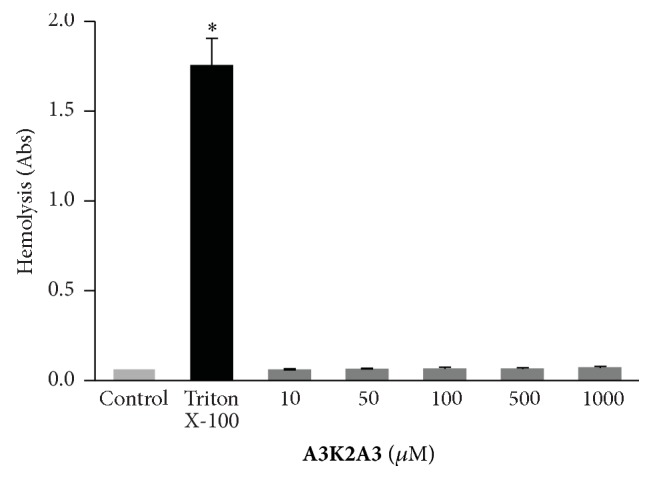
Hemolytic properties of** A3K2A3 **on human red blood cells. Triton X-100 was used as a positive control. Results are expressed as mean ± SD of at least three independent experiments. ^*∗*^*p* ≤ 0.05, significant difference of each group from control.

In order to check the toxicity of the compounds the hemolytic potential was assessed. Red blood cells were incubated in the presence of different concentrations of** A3K2A3** for 3 h. After incubation, the highest concentration tested (1000.0 *μ*M) did not induce hemolysis (Figure 5).

### 3.5. The Combination of** A3K2A3 **with Benznidazole, Ketoconazole, or Fluconazole Induces Effects on* Trypanosoma cruzi* and LLCMK_2_ Cells

In order to improve the activity of** A3K2A3** and get synergistic effect combinations between this compound and benznidazole, ketoconazole, or fluconazole were performed. These combinations were tested on epimastigotes, trypomastigotes, and LLCMK_2_ cells.

**Figure 6:**
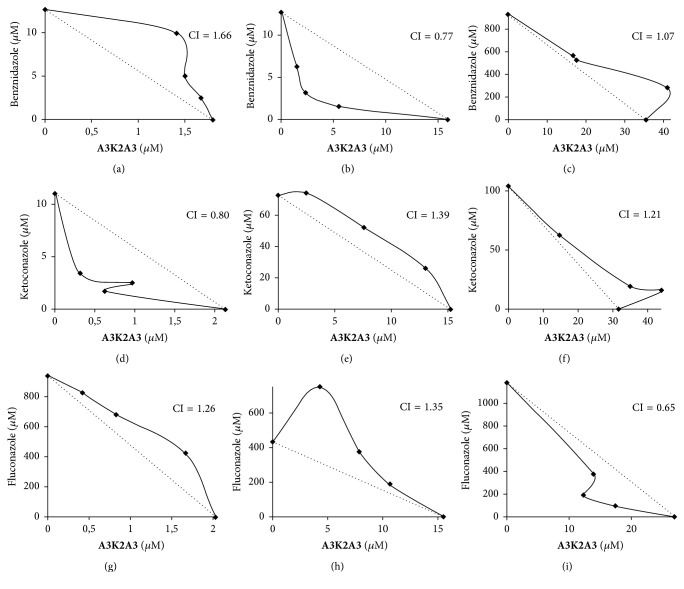
Isobolograms of drugs combinations. Effect of the combination of** A3K2A3** and benznidazole against epimastigotes (a), trypomastigotes (b), and LLCMK_2_ cells (c). Effect of the combination of** A3K2A3** and ketoconazole against epimastigotes (d), trypomastigotes (e), and LLCMK_2_ cells (f). Effect of the combination of** A3K2A3** and fluconazole against epimastigotes (g), trypomastigotes (h), and LLCMK_2_ cells (i). The dotted lines correspond to the additivity effect. Points below the line indicate a synergistic effect. Points above the line indicate an antagonistic effect. The experiment was repeated three times. The points show median values.

The combination of benznidazole and** A3K2A3** despite showing antagonistic effect on epimastigotes (CI of 1.66) (Figure 6(a)) and on LLCMK_2_ cells (CI of 1.07) (Figure 6(c)) showed synergistic effect on trypomastigotes (CI of 0.77) (Figure 6(b)).

The combination of ketoconazole and** A3K2A3** showed synergistic effect on epimastigotes (CI of 0.80) (Figure 6(d)) and antagonistic effect on LLCMK_2_ cells (CI of 1.21) (Figure 6(f)) and on trypomastigotes (CI of 1.39) (Figure 6(e)).

The combination of fluconazole and** A3K2A3** also showed antagonistic effect on epimastigotes (CI of 1.26) and trypomastigotes (CI of 1.35) (Figures 6(g) and 6(h)), but on LLCMK_2_ cells the effect is synergistic (CI of 0.65) (Figure 6(i)).

### 3.6. **A3K2A3** Induces Alterations in* Trypanosoma cruzi* Morphology and Ultrastructure

**Figure 7:**
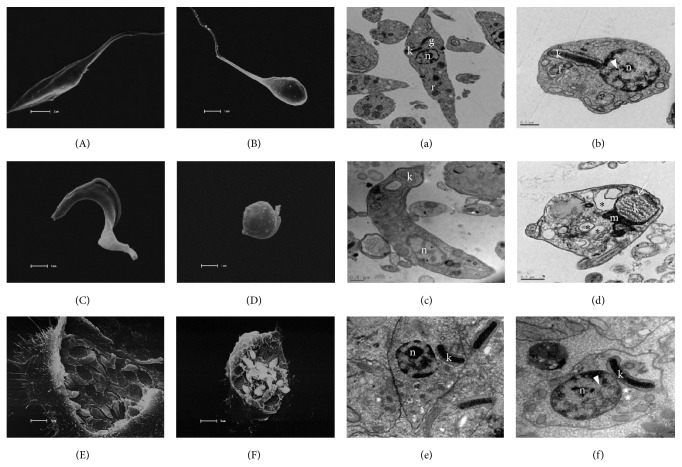
Scanning electron microscopy and transmission electron microscopy of epimastigote forms of* Trypanosoma cruzi* treated with** A3K2A3** for 72 h at 28°C and trypomastigotes and amastigotes forms of* Trypanosoma cruzi* treated with** A3K2A3** for 24 h at 37°C. Uppercase letters correspond to scanning electron microscopy and lowercase letters correspond to electronic transmission microscopy. (A, a) Untreated epimastigotes, (C, c) untreated trypomastigotes, and (E, e) untreated amastigotes exhibited normal morphology and organelles. (B, b) Epimastigotes treated with IC_50_ of** A3K2A3** exhibited reduction and rounding of the cell volume and nuclear disorganization (white arrow head). (D, d) Trypomastigotes treated with EC_50_ of** A3K2A3** exhibited reduction and rounding of the cell volume and concentric myelin-like membrane structures (black arrow), autophagosome-like structures (asterisk), and mitochondrial swelling (white arrow). (F, f) Amastigotes treated with IC_50_ of** A3K2A3** exhibited normal morphology and lipid-storage bodies (star) and nuclear disorganization (white arrow head). Bars = 5 *µ*m (E, F); bars = 2 *µ*m (A, a); bars = 1 *µ*m (B, C, D); bars = 0.5 *µ*m (b, c, d); bars = 0.2 *µ*m (e, f). n, nucleus; r, reservosomes; m, mitochondrion; k, kinetoplast; g, Golgi complex; f, flagellum.

Ultrastructural and morphological alterations induced by treatment with** A3K2A3** were evaluated by transmission electron microscopy and scanning electron microscopy, respectively. The parasites treated with** A3K2A3** presented serious structural changes in the three evolutionary forms (Figure 7). The morphological changes were more evidenced in epimastigotes and trypomastigotes (Figures 7(B) and 7(D)). Untreated cells showed normal organelles (Figures 7(a), 7(c), and 7(e)). Major ultrastructural changes were quantified, and it was observed that the compound promoted the same changes in three evolutionary forms of* T. cruzi* (Table 1). The nucleus, the autophagosome-like structures, the mitochondrion, the cytoplasmatic membrane, and the Golgi complex were the structures most affected. We also observed the formation of myelin-like figures and a large increase in the number of autophagosome-like structures and lipid-storage bodies (Figures 7(b), 7(d), and 7(f)). The morphology of the parasites treated with the compound showed alterations mainly on epimastigote and trypomastigote forms. The main morphological alterations induced by treatment with this compound were rounding and reduction of the cellular body and loss of flagellum in both parasitic forms (Figures 7(B) and 7(D)). Amastigote forms showed no significant morphological changes (Figure 7(F)). However, a reduction in the number of intracellular amastigotes was observed (Figure 7(F)).

## 4. Discussion

Dibenzylideneacetones are compounds characterized by a broad spectrum of biological activity and exhibit important biological activities against several infections [22–24]. The dibenzylideneacetone** A3K2A3** evaluated in this study has two nitro groups attached directly to an aromatic ring system [26, 32].

**A3K2A3** exhibited strong anti-*Trypanosoma cruzi* activity against the main forms of the parasite. This compound showed better activity against the parasites than did the reference drug, benznidazole (IC_50 epi_: 6.5 *μ*M; EC_50 trypo_: 34.5 *μ*M; IC_50 ama_: 19.2 *μ*M) [10]. These results are especially interesting because it demonstrates that this compound can be promising chemotherapeutic agent against* T. cruzi*.

The presence of two nitro groups in** A3K2A3** can justify its good activity against this protozoan, since the nitro group is essential for the action of the drug. These results are consistent with the literature, which showed that nitroreductase enzymes, found in trypanosomes and absent in mammals [33–36], may activate NO_2_-containing drugs into a more active form [26]. Nitroreductase enzymes have two categories, the type I NTRs (oxygen-insensitive) which perform reduction of two electrons and do not result in the production of reactive oxygen species (ROS), and type II NTRs (sensitive to oxygen) which perform reduction of an electron leading to the production of an unstable nitro-radical that in the presence of oxygen results in the production of superoxide anions [37–40].

When the compound was tested on trypomastigotes at 4°C, a drastic reduction of activity of the** A3K2A3** compound was observed. This reduction mediated by the low temperature was also analyzed in other compounds [41] and should be related to the compromised enzymes activity of the parasite. The literature shows that low temperatures affect the enzymatic activity because, from a certain temperature, reaction rate decreases sharply [42].** A3K2A3** is nitro compound, presenting in its chemical structure the presence of nitro groups attached directly to an aromatic ring [26]. The literature reports that the bioactivity of nitro compounds is related to its reduction, acting as a prodrug [32]. In fact, a metabolomic study of** A3K2A3 **performed by Din et al. (2016) disclosed that the action of* T. cruzi* can modify the** A3K2A3 **compound in the result of metabolism. This investigation confirmed that enzymes of* T. cruzi* play a pivotal role in drug activation [43], which may explain the drastic reduction of activity of the** A3K2A3** compound in trypomastigotes at 4°C.

An important criterion in the search for compounds active against* T. cruzi* with therapeutic potential is that they are not toxic to the mammalian host cells. According to Garratty [44] some drugs can induce immune hemolytic anemia (DIIHA) so it is important to check the hemolytic capacity of the compounds.** A3K2A3** showed no hemolytic activity and high selectivity index for the three evolutionary forms, epimastigotes (SI_**A**3**K**2**A**3_: 15.77), trypomastigotes (SI_**A**3**K**2**A**3_: 2.01), and amastigotes (SI_**A**3**K**2**A**3_: 27.16). The cytotoxicity of this compound also had been evaluated against macrophages J774A1 and LLCMK_2_ cells and results showed that the compound was more toxic to* Leishmania amazonensis* and* T. cruzi *than for mammalian cells [26]. Aher et al. [22] also revealed no toxicity of dibenzylideneacetones.

Combination of drugs is an important way to improve the therapeutic efficacy. Generally, the combinations are employed in order to enable a synergetic effect, reducing toxicity, the treatment time, and development of resistance [45, 46]. Combination of drugs is a promising strategy for the development of new treatments for Chagas disease [47]. In this context, we sought to evaluate the combination of** A3K2A3** with the standard drug benznidazole and the azoles, ketoconazole and fluconazole. Azole compounds have been shown to be effective in blocking the proliferation of protozoa parasites such as* L. tropica*,* L. mexicana*, and* T. cruzi*, both in vitro and in vivo [48, 49].

The synergistic activity of ketoconazole and benznidazole interaction with other compounds in* T. cruzi* has already been demonstrated [9, 20, 47, 48, 50, 51]. Fluconazole has shown synergistic effect against other protozoa, when combined with other drugs [52].** A3K2A3** was effective in combination with benznidazole against trypomastigotes and in combination with ketoconazole against epimastigotes. These combinations also exhibited antagonistic effect on LLCMK_2_ cells. The combination of the compound with fluconazole was not satisfactory. Fluconazole despite having been shown to be active in vitro against* T. cruzi* [6] cannot stop the progression of the disease [40, 53].

Recent research shows that some synthetic compounds promote cellular disorganization in* L. infantum* affecting mainly the nucleus, mitochondria, and kinetoplast, with frequent disruption of the nuclear membrane, loss of cellular integrity, and accumulation of bodies lipids [54]. In* T. cruzi*, ultrastructural changes as the formation of myelin figures, autophagosomes-like vacuoles, disruption of the Golgi complex, and mitochondria enlargement were observed in response to treatment with inhibitors of sterol biosynthesis [55, 56].

In this study, the analysis performed by SEM showed that treatment with** A3K2A3** in epimastigotes and trypomastigotes led to morphological changes that affect the flagellum, reduction of the volume, and rounding of the cell. In amastigotes there was no change in morphology. TEM showed that the compound structurally affects mainly the nucleus, mitochondrion, plasma membrane, and Golgi complex in the three parasitic forms. The increased number of autophagosome-like structures also was observed. The presence of these structures could imply the existence of process of autophagy.

Intense cytoplasmic vacuolization and structural disorganization of the parasite cell, together with mitochondrial swelling, were also observed after treatment with other compounds in* T. cruzi* [10, 57, 58]. Mitochondrion of* T. cruzi* presents unique functional and structural features, which are remarkably different from mammalian mitochondria. These features make this organelle an exceptionally attractive chemotherapeutic target [41].

Altogether, our findings support the possibility that different mechanisms may be related to the action of the** A3K2A3** against* T. cruzi*. Despite the fact that many of the specific targets of drugs used against* T. cruzi* are known, it is reasonable to suppose that one compound could act on more than one target with independent or combined action, as well as in a single preferred pathway.

## 5. Conclusions

In conclusion, the present study demonstrated the strong trypanocidal activity of** A3K2A3 **on the three forms of* T. cruzi*. These effects were marked by morphological and ultrastructural changes in this parasite. The data suggest that** A3K2A3** might be promising compound for the treatment of Chagas disease because its action is active in more than one target on* T. cruzi*. In addition, the combination of** A3K2A3** with benznidazole was the most promising, because it increased the effectiveness against trypomastigotes, the clinically relevant form of* T. cruzi*. Therefore, our results support additional in vivo studies using this drug combination in infected animals.

## Figures and Tables

**Table 1:** Summary of damage on *Trypanosoma cruzi* observed by transmission electron microscopy, after 24 h exposure to compound **A3K2A3**.

Structure	Damage (%)^a,b^					
	Epimastigotes		Trypomastigotes		Amastigotes	
	Untreated	IC_50_	Untreated	EC_50_	Untreated	IC_50_
Mitochondrion	08	68	02	75	06	65
Plasmatic membrane	08	76	01	50	02	28
Cell division	04	36	—	—	06	19
Nucleus	12	84	08	65	02	19
Autophagic vacuoles	04	62	09	80	06	14
Lipid vacuoles	04	20	11	40	00	02
Golgi complex	00	48	00	70	01	05
Myelin figures	00	04	00	05	00	01

^a^For each compound, 100 parasites were analyzed.

^b^Damage frequency: 0% = no damage; 100% = maximum damage.
